# Deacylcynaropicrin Inhibits RANKL-Induced Osteoclastogenesis by Inhibiting NF-κB and MAPK and Promoting M2 Polarization of Macrophages

**DOI:** 10.3389/fphar.2019.00599

**Published:** 2019-06-07

**Authors:** Zhikun Li, Xiaodong Zhu, Ruijun Xu, Yi Wang, Ruixi Hu, Wei Xu

**Affiliations:** Department of Orthopaedics, TongRen Hospital, School of Medicine, Shanghai JiaoTong University, Shanghai, China

**Keywords:** deacylcynaropicrin, osteoclastogenesis, MAPK, NF-κB, macrophages

## Abstract

Inflammation can promote the maturity of osteoclasts and bone resorption in many bone disease such as osteoporosis and arthritis. Here, we aimed to investigate the inhibitory effects of deacylcynaropicrin (DAC) on osteoclastogenesis and bone resorption induced by RANKL. Bone-marrow-derived macrophages were used for assessing the influence of DAC on polarization of macrophages and osteoclastogenesis *in vitro*. Inducible nitric oxide synthase (iNOS) and CD206, as well as osteoclastogenesis markers, nuclear factor of activated T-cells 1 (NFATc1), and c-Fos, were qualitatively analyzed by immunofluorescence, flow cytometry, reverse transcription polymerase chain reaction, and Western blotting. The results showed that DAC significantly inhibited osteoclastogenesis by suppressing the expression levels of c-Fos and NFATc1 through nuclear factor-κB, c-Jun N-terminal kinase (JNK), and Akt pathway. Moreover, immunohistochemistry and enzyme-linked immunosorbent assays showed that DAC reduced the release of tumor necrosis factor-α, interleukin (IL)-1β, and IL-6 *in vivo*. Finally, DAC also promoted macrophage polarization from M1 to M2 types. In conclusion, these results demonstrated that DAC suppressed RANKL-induced inflammation and osteoclastogenesis and therefore it can be used as a potential treatment for osteoporosis, arthritis, osteolysis, and aseptic loosening of artificial prostheses.

## Introduction

Overactivation of osteoclasts can be observed in many inflammatory bone diseases such as arthritis and osteoporosis, which bring a heavy burden on the health and economy of people (Hopkins et al., 2016; Ha et al., 2017). In the microenvironment of bone, the activated lymphocytes and macrophages can release many inflammatory cytokines including TNF-α and IL-1β, which can recruit monocytes in the bone marrow and promote the expression of receptor activator of nuclear factor kappa-B (NF-κB) ligand (RANKL) and inhibition of osteoprotegerin (OPG) expression in osteoblasts, resulting in osteoclastogenesis and bone resorption (Nich et al., 2011; Lin et al., 2014; Zhai et al., 2014). RANKL can bind with the RANK on the surface of monocytes, the precursors of osteoclasts, and activate the downstream factor TRAF6 and NF-κB to promote the expressions of osteoclastogenesis-associated genes including NFATc1 and c-fos (Takayanagi et al., 2002). Given the importance of osteoclasts in osteoporosis and arthritis, treatments targeting osteoclast differentiation and function provide a reasonable solution for mitigating osteolytic and pathological bone loss.

Previous literature reported that adult tissue-resident macrophages in liver (Kupffer cells), brain (microglia), epidermis (Langerhans cells), and lung (alveolar macrophages) originate from a Tie2(+) (also known as Tek) cellular pathway generating Csf1r(+) erythro-myeloid progenitors (EMPs) distinct from HSCs (Gomez Perdiguero et al., 2015). As an important performer of the innate immune system, macrophages can regulate the immune microenvironment *in vivo* by secreting a variety of cytokines. Macrophages can be polarized to M1 and M2 after being stimulated differently. M1-type macrophages are pro-inflammatory and secrete various inflammatory cytokines such as TNF-α and IL-1β. M2 macrophages are anti-inflammatory and reduce the level of inflammation in the bone marrow microenvironment (Trial et al., 2013; Saqib et al., 2018). It has been reported that bone absorption and osteogenesis are closely related to the bone marrow immune microenvironment (Lechner et al., 2018; Walsh et al., 2018; Gruber, 2019). TNF-α-induced osteoclast recruitment and differentiation may be the core of the pathogenesis of inflammatory bone diseases (Espirito Santo et al., 2015). Therefore, osteoclastogenesis can also be regarded as an inflammatory response.

In fact, several agents that target the function of osteoclasts have been studied in prior decades, including nitrogen-containing bisphosphonates, erythromycin, strontium ranelate, and denosumab (Looney et al., 2006; Ren et al., 2009; Qu et al., 2013; Liu et al., 2014). However, the therapeutic effect of bisphosphonates and other anticatabolic methods would be compromised after a long period of usage (Schwarz et al., 2000; Schwarz, 2008). Researchers are increasingly interested in alternative materials that can be used for prevention and therapy of osteoporosis. Deacylcynaropicrin (DAC), isolated from the aerial parts of *Cyclolepis genistoides* or the flowers of *Hemisteptia lyrata* Bunge, has been demonstrated to inhibit carrageenan-induced inflammation (Ha et al., 2003; Sosa et al., 2011). Other research revealed that DAC presents moderate antibacterial activity against *Staphylococcus aureus* and its cytotoxic activities against cancer cells (Ha et al., 2003; Shakeri et al., 2018). However, no studies have evaluated the effects of DAC on the osteoclastogenesis and prevention of inflammatory osteolysis.

Accordingly, in this study, we examined the effect of DAC on LPS-induced inflammation and RANKL-induced osteoclastogenesis *in vitro* and on LPS-induced inflammatory osteolysis of mouse calvaria. Furthermore, the activation of NF-κB and JNK during osteoclastogenesis with or without DAC was evaluated to reveal its inhibitory mechanism. Our findings provided important insights into the potential applications of DAC in the prevention and treatment of inflammatory osteolysis.

## Materials and Methods

### Reagents

DAC (CAS No. 31565-50-1, MOLBASE Biotechnology Co. Ltd, Shanghai, China) was dissolved in dimethyl sulfoxide without exposure to light and stored at −20°C. Dulbecco’s modified Eagle’s medium (DMEM) was provided by Hyclone (Logan, UT, USA). Penicillin–streptomycin solution, trypsin–ethylenediaminetetraacetic acid (EDTA) solution (0.25%), and fetal bovine serum (FBS) were obtained from Gibco (Gaithersburg, MD, USA). Recombinant mouse RANKL and macrophage-colony-stimulating factor (M-CSF) were provided by PeproTech (Rocky Hill, NJ, USA). A tartrate-resistant acid phosphatase (TRAP) staining kit, Triton X-100, and 4,6-diamidine-2-phenylindole dihydrochloride (DAPI) were purchased from Sigma-Aldrich. A Cell Counting Kit-8 (CCK-8) was provided by Dojindo Molecular Technology Inc. (Kumamoto, Japan). Acti-stain 555 fluorescent phalloidin was obtained from Cyto-skeleton Inc. (Denver, CO, USA). The specific primary antibodies and secondary antibodies used in the experiments were provided by Cell Signaling Technology (Danvers, MA, USA).

### Isolation of Mouse-Bone-Marrow-Derived Macrophages and Cell Culture

Primary bone-marrow-derived macrophages (BMMs) were isolated from bone marrow aspirates from 6- to 8-week-old male C57BL/6 mice as described previously (Guan et al., 2015; Oh et al., 2015; Wang et al., 2017). Briefly, bone marrow cells were isolated from aspirates of tibiae and femurs, followed by incubation in 10-cm culture dishes containing α-MEM supplemented with 10% (v/v) FBS and 30 ng/ml M-CSF at 37°C in a humidified atmosphere (5% CO_2_, 95% air) for 12–24 h. The suspension of nonadherent cells was then collected and reseeded in another 10-cm dish for further culture. After 3 days of incubation, medium containing nonadherent cells (e.g., hemocytes and lymphocytes) and impurities was then discarded, and adherent cells were used as BMMs. When the cells reached 90% confluence, BMMs were harvested by trypsin digestion and seeded into culture plates or dishes for further experiments.

### Cell Viability Assays

Varying concentrations of DAC were selected to evaluate cell viability. BMMs were seeded at a density of 8,000 cells/well (in triplicate) and incubated in 96-well plates for 24 h. Cells were then treated with vehicle and different concentrations of DAC (1.25, 2.5, 5, 10, 20, 40, or 80 μM) for 24, 48, 72, or 96 h. Culture media (100 μl/well) were changed at 48 h. At the time points, the media were replaced with 100 μl/well FBS-free cultural medium containing 10 μl (10%) of CCK-8 solution. Then, the plates were incubated for 2 h at 37°C in the dark. Next, the absorbance was detected at a wavelength of 450 nm (630 nm as a reference) on a Bio-Tek Synergy HT spectrophotometer. The experiment was independently repeated three times.

### RANKL-Induced Osteoclastogenesis *In Vitro*

A total of 1 × 10^4^ BMMs/well were cultured in 96-well plates and incubated with 30 ng/ml M-CSF, 100 ng/ml RANKL, and different noncytotoxic concentrations of DAC (2.5, 5, or 10 μM). After 7 days, cells were fixed with 4% paraformaldehyde for 30 s and then stained using a TRAP kit according to the manufacturer’s instructions. TRAP-positive multinucleated cells (MNCs; nuclei > 3) were counted as osteoclasts and measured using Image-Pro Plus 6.0 software (Wu et al., 2012; Ouyang et al., 2014).

### F-actin Ring Formation Assays and Bone Resorption Pit Assays

For F-actin ring fluorescence staining, 8 × 10^4^ BMMs/dish were seeded onto 35-mm confocal culture dishes. Twenty-four hours later, medium was replaced with medium containing M-CSF and RANKL with or without DAC (5 or 10 μM), and cells were cultured for 7 days. The cells were then fixed with 4% paraformaldehyde for 15 min and permeabilized with 0.1% (v/v) Triton X-100 for 5 min. After washing with PBS three times, cells were stained for F-actin using rhodamine-conjugated phalloidin (1:100; Invitrogen Life Technologies, USA) diluted in 0.2% w/v bovine serum albumin (BSA) in PBS at 37°C for 1 h. Cells were then washed with PBS, and nuclei were stained using DAPI for 5 min at room temperature. An LSM5 confocal microscope (Carl Zeiss, Oberkochen, Germany) was used to detect the formed F-actin ring.

Bone resorption pit assays were performed using Corning Osteo Assay Surface 24-Well Plates (cat. no. #3987; Corning, NY, USA) coated with hydroxylapatite. Next, 8 × 10^4^ BMMs/well were seeded and cultured with 30 ng/mL M-CSF and 100 ng/mL RANKL with or without DAC (0, 5, or 10 μM) for 7 days. Von Kossa staining was used to increase contrast between pits and the surface coating, and visual enumeration of pits was carried out using a microscope or analytical software. The number of resorption pits (/mm^2^) was counted, and the percentage of the resorption area was measured. Three replicates were performed per well, and the experiment was repeated independently three times.

### Immunofluorescent Staining

BMMs were seeded onto confocal culture dishes (1 × 10^5^ cells/well) and incubated with vehicle and 5 or 10 μM DAC. After culturing for 3 days, 4% paraformaldehyde in PBS was used to fix the cells for 15 min at room temperature. The cells were then washed three times in PBS before permeabilized by 0.1% Triton X-100. Nonspecific binding sites were blocked with 10% BSA in PBS for 1 h. Primary monoclonal antibodies for iNOS (1:100; Abcam, Cambridge, UK) and CD206 (1:100; Abcam) were incubated in PBS containing 1% BSA at 4°C overnight. Cells were washed three times in PBS. Cells were incubated with goat anti-rabbit Alexa Fluor 488 (1:200) and goat anti-rabbit Alexa Fluor 594 (1:200; Abcam) at room temperature for 2 h as secondary antibodies. Cell nuclei were stained with DAPI for 15 min. The cells were then washed three times in PBS. Images were processed on an LSM5 confocal microscope (Carl Zeiss, Oberkochen, Germany).

### Flow Cytometry

The inflammatory exudates were obtained and analyzed by flow cytometry according to the method described by Vasconcelos et al. (2013). For flow cytometry analysis, BMMs were seeded into 6-cm culture dishes (5 × 10^5^ cells/well) with or without DAC (0 or 10 μM). The expression levels of macrophage cell subpopulation markers CCR7 (M1) and CD206 (M2) were analyzed by flow cytometry to evaluate the different phenotypes. After 3 days of culture, cells were scraped from the plates, centrifuged, and resuspended in 1% BSA for 30 min at room temperature to block nonspecific antigens. Then, cells were incubated with fluorescein isothiocyanate (FITC)-conjugated anti-mouse F4/80, allophycocyanin-conjugated CCR7, and phycoerythrin-conjugated CD206 for 30 min at room temperature. FITC-conjugated rat IgG2a,κ, APC-conjugated rat IgG2a,κ, and 229 PE-conjugated rat IgG2a,κ were used as isotype controls. All antibodies used for flow cytometry were purchased from eBioscience. All antibodies used for flow cytometry were purchased from BD Pharmingen. After washing twice with PBS, cells were resuspended in 1% BSA and analyzed on a Guava flow cytometer (Millipore, USA). Data were analyzed using Guava Soft 3.1.1.

### LPS-Induced Mouse Calvarial Osteolysis Model

All experiments were approved by the Ethics Committee of Shanghai Tongren Hospital affiliated with Shanghai Jiao Tong University School of Medicine. Animal care and experiments were conducted in accordance with guidelines and procedures authorized by the Animal Care and Use Committee of Shanghai Jiao Tong University School of Medicine. As previously described (Zhu et al., 2016), a mouse calvarial osteolysis model was established in 8-week-old male C57BL/6J mice obtained from the Experimental Animal Center of Shanghai Tongren Hospital to investigate the preventive and therapeutic effects of DAC on LPS-induced inflammatory osteolysis. Briefly, 40 healthy mice were randomly divided into four groups, i.e., the sham operation group (100 μl/day PBS), control group (100 μl/day PBS), low-dose DAC group (10 mg/kg/day DAC), high-dose DAC intervention group (20 mg/kg/day DAC). After abdominal anesthesia by 1% pentobarbital, 100 μg LPS or 100 μl PBS was daily injected onto the middle surface of calvaria as previously reported for 7 days (Kimura et al., 2012; Saeed et al., 2016). Animals in the sham group underwent sham surgery only. Mice in the low- and high-dose DAC groups were injected intraperitoneally with DAC. Mice in the sham and vehicle groups received PBS daily by intraperitoneal injection. All mice survived the experimental period and were sacrificed on 14 days. The calvaria were then excised and harvested for micro-computed tomography (micro-CT), histological analysis, and molecular analysis.

### Micro-CT Scanning

Paraformaldehyde-fixed calvaria were scanned using a high-resolution micro-CT (μCT 80; SCANCO Medical AG, Bassersdorf, Switzerland). The scanning protocol was set at an isometric resolution of 9 mm, and X-ray energy settings were 70 kV, 114 mA, and 8 W for assessing osteolysis and the therapeutic effects of DAC. The three-dimensional images were reconstructed with thresholding of 200 for each sample. The bone mineral density (BMD) and bone volume/tissue volume (BV/TV) in a square-shaped region of interest (ROI; 3.5 mm × 3.5 mm) were measured using the software provided with the micro-CT system for quantitative analysis of scan results as previously reported (Wedemeyer et al., 2007). The number and percentage of porosity in the ROI were analyzed using Image-Pro Plus 6.0 software.

### Histological Staining and Immunohistochemical Analysis

After micro-CT imaging, the calvaria were decalcified in 10% EDTA (pH 7.4) for 4 weeks and embedded in paraffin for histological sections. The sections were stained with hematoxylin and eosin (H&E) and TRAP stain. The specimens were photographed under a high-quality optical microscope (Leica DM4000B) connected to computers. The number of TRAP stain-positive osteoclasts was counted and normalized to the bone area. The bone area was measured using Image-Pro Plus 6.0 software.

### Enzyme-Linked Immunosorbent Assay

As previously described (Nich et al., 2011; Zhai et al., 2014; Shao et al., 2015), BMMs were incubated in 12-well plates with each well containing 1 ml of DMEM with 1% penicillin and streptomycin at 37°C with 5% CO_2_ for 24 h. The culture media were then collected and stored at −80°C for enzyme-linked immunosorbent assay (ELISA) analysis of TNF-α (eBioscience, San Diego, CA, USA), IL-1β (eBioscience), and IL-6 (eBioscience) secretion. The absorbance was detected at a wavelength of 490 nm on a Bio-Tek Synergy HT spectrophotometer according to the manufacturer’s instructions. Three independent experiments were performed.

### Total Protein Extraction and Western Blotting

The BMMs were lysed with radio immunoprecipitation assay (RIPA) buffer to extract proteins. Protein concentrations were determined using a BCA Assay Kit (Thermo Fisher Scientific). Total proteins were stored at −80°C. Protein extracts (25 mg) were separated on sodium dodecyl sulfate polyacrylamide gels and transferred to polyvinylidene difluoride membranes (Millipore). The membranes were incubated with primary antibodies at 4°C overnight. After secondary antibodies were added for 1 h at room temperature, the protein bands were detected by Odyssey V3.0 image scanning (Li-COR Inc., Lincoln, NE, USA). The intensity of each band was analyzed using Image J software.

### Statistical Analysis

The data from each experiment are presented as means ± standard deviations from at least three independent experiments. Results were analyzed using Student’s *t* tests or analysis of variance, followed by Dunnett’s test for *post hoc* analysis using SPSS 20.0 software (SPSS Inc., Chicago, IL, USA). Differences with a *p* value of less than 0.05 indicated statistical significance between groups.

## Results

### Effects of DAC on the Proliferation of BMMs

**Figure 1:**
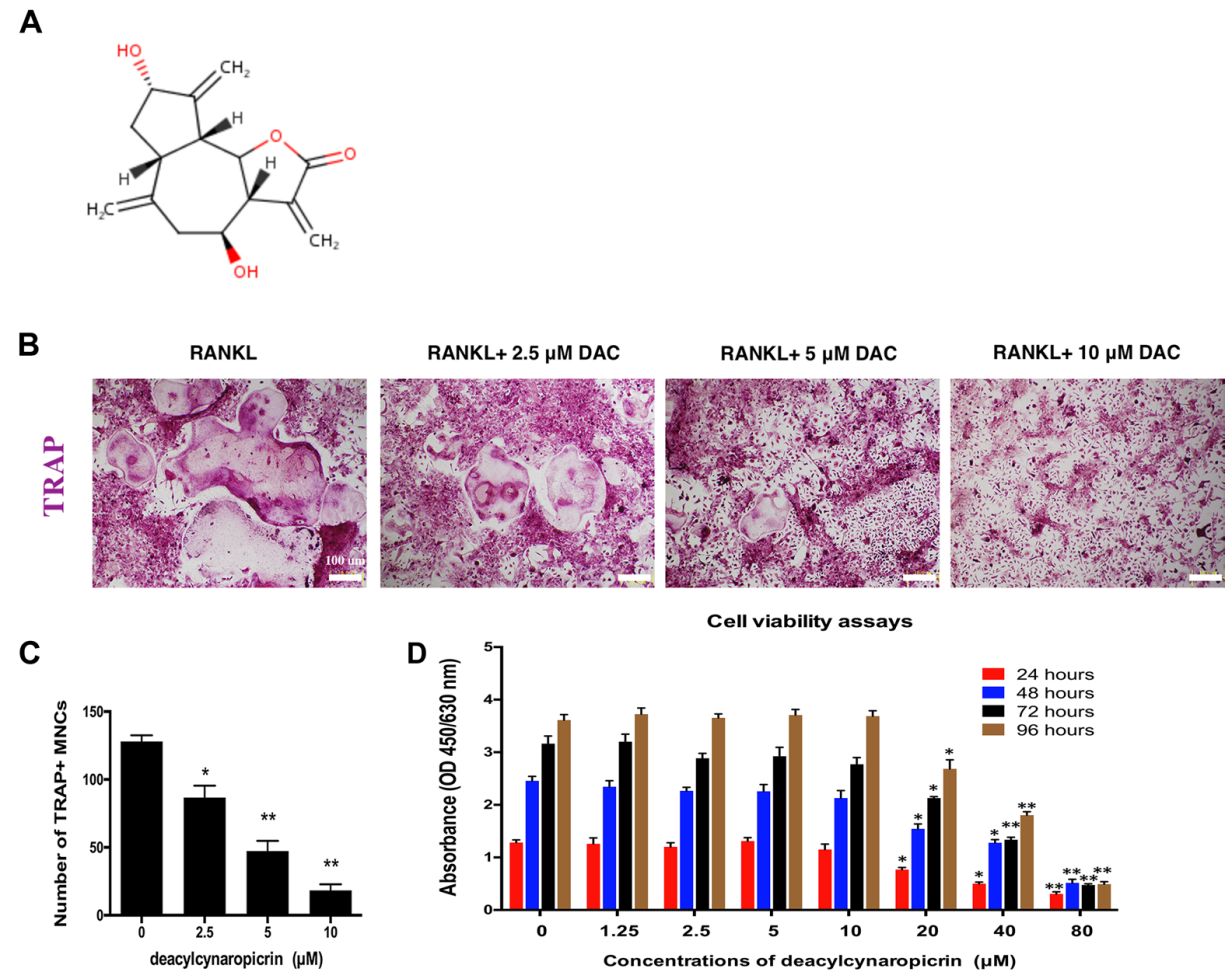
DAC inhibited RANKL-induced osteoclastogenesis. **(A)** Chemical structure of DAC. **(B)** BMMs were induced with M-CSF, RANKL, and 0, 5, or 10 μM DAC and assessed by TRAP staining. **(C)** The number of TRAP-positive MNCs was determined. **(D)** Cell viability following DAC stimulation was measured by CCK-8 assays at 24, 48, 72, and 96 h. Results were analyzed using ANOVA followed by Dunnett’s test for *post hoc* analysis. *p < 0.05, **p < 0.01.

The chemical structure of DAC is shown in **Figure 1A**. First, the effect of DAC on the cell viability of BMMs was evaluated by CCK-8 assays. The results revealed that the OD values in groups treated with DAC less than 20 μM were not significantly different at 24, 48, 72, and 96 h (*p* > 0.05). However, the OD values in groups treated with 20, 40, or 80 μM DAC were dramatically lower than those in groups lower than 20 μM DAC at all time points (*p* < 0.01 or *p* < 0.05) (**Figure 1D**). Therefore, 5 and 10 μM DAC were used as low and high concentrations, respectively, to perform further experiments without cytotoxicity.

### Inhibitory Effects of DAC on Osteoclastogenesis *In Vitro*

In osteoclastogenesis assays, TRAP staining revealed that RANKL remarkably induced the formation of multiple nucleus cells (MNCs) regarded as mature osteoclasts (**Figure 1B**). The number of MNCs in the TRAP-positive stained area in DAC-treated groups were significantly decreased by a dose-dependent manner (*p* < 0.05, c), which demonstrated that DAC can inhibit RANKL-induced formation of osteoclasts from BMMs *in vitro*.

**Figure 2:**
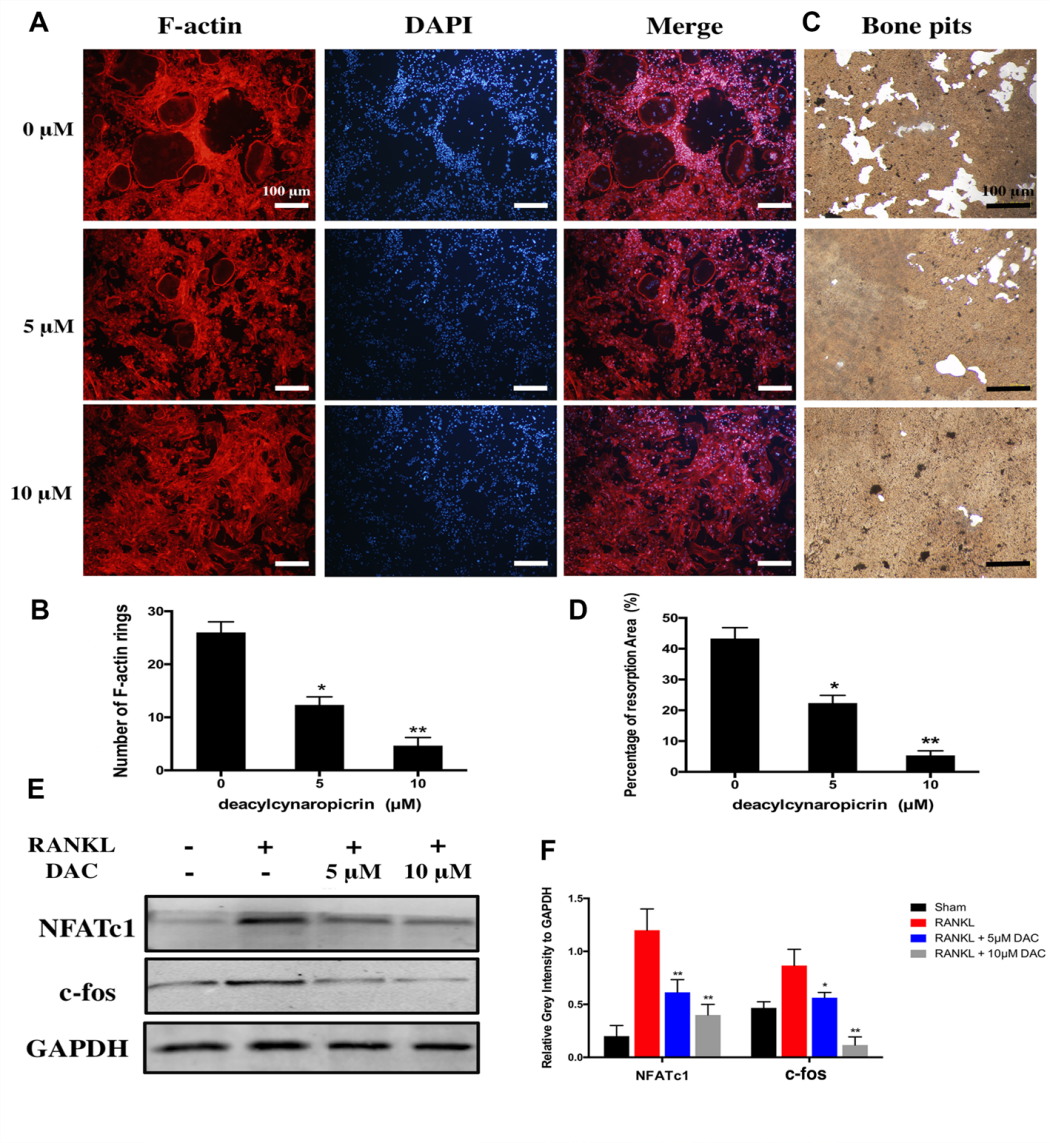
Evaluation of the effects of DAC on the formation of F-actin rings in osteoclasts and hydroxylapatite resorption by fluorescence staining and hydroxylapatite-coated plates. **(A)** BMMs were incubated with M-CSF and RANKL as well as 0, 5, or 10 μM DAC and then stained with 555 fluorescent phalloidin and DAPI. **(B)** Number of F-actin rings in osteoclasts. **(C)** Bone resorption was assessed using hydroxylapatite-coated 24-well plates with Von Kossa staining. **(D)** Percentage of resorption area. **(E)** The NFATc1 and c-fos were evaluated during RANKL-induced osteoclastogenesis by Western blot. **(F)** Relative gray intensity to GAPDH was calculated. Results were analyzed using ANOVA followed by Dunnett’s test for *post hoc* analysis. *p < 0.05, **p < 0.01.

Moreover, given that F-actin rings play a critical role in bone resorption by osteoclasts, we further investigated the effects of DAC on the function of osteoclasts including the formation of F-actin rings and bone resorption. The results suggested that the number of F-actin ring in the RANKL-induced group was remarkably more than that in the 5 μM (*p* < 0.05) or 10 μM (*p* < 0.01) DAC-treated groups (**Figure 2A and B**). In addition, hydroxylapatite resorption pits by mature osteoclasts in the RANKL-induced group were apparently observed (**Figure 2C**). Nevertheless, the number of resorption pits was decreased in DAC-treated BMMs (*p* < 0.05, **Figure 2D**).

Then, the expression of NFATc1 and c-fos, which are the critical mediators of osteoclastogenesis, were detected by immunoblot. The NFATc1 and c-fos in RANKL-induced osteoclasts were upregulated compared with control. However, consistent with above results, NFATc1 and c-fos were significantly inhibited in DAC-treated cells (*p* < 0.05, **Figure 2E and F**).

Taken together, DAC dramatically inhibited the function of osteoclasts as well *via* decreasing the formation of F-actin rings and hydroxylapatite resorptions in a concentration-dependent manner.

### DAC Inhibits the LPS-Induced Secretions of Inflammatory Cytokines

**Figure 3:**
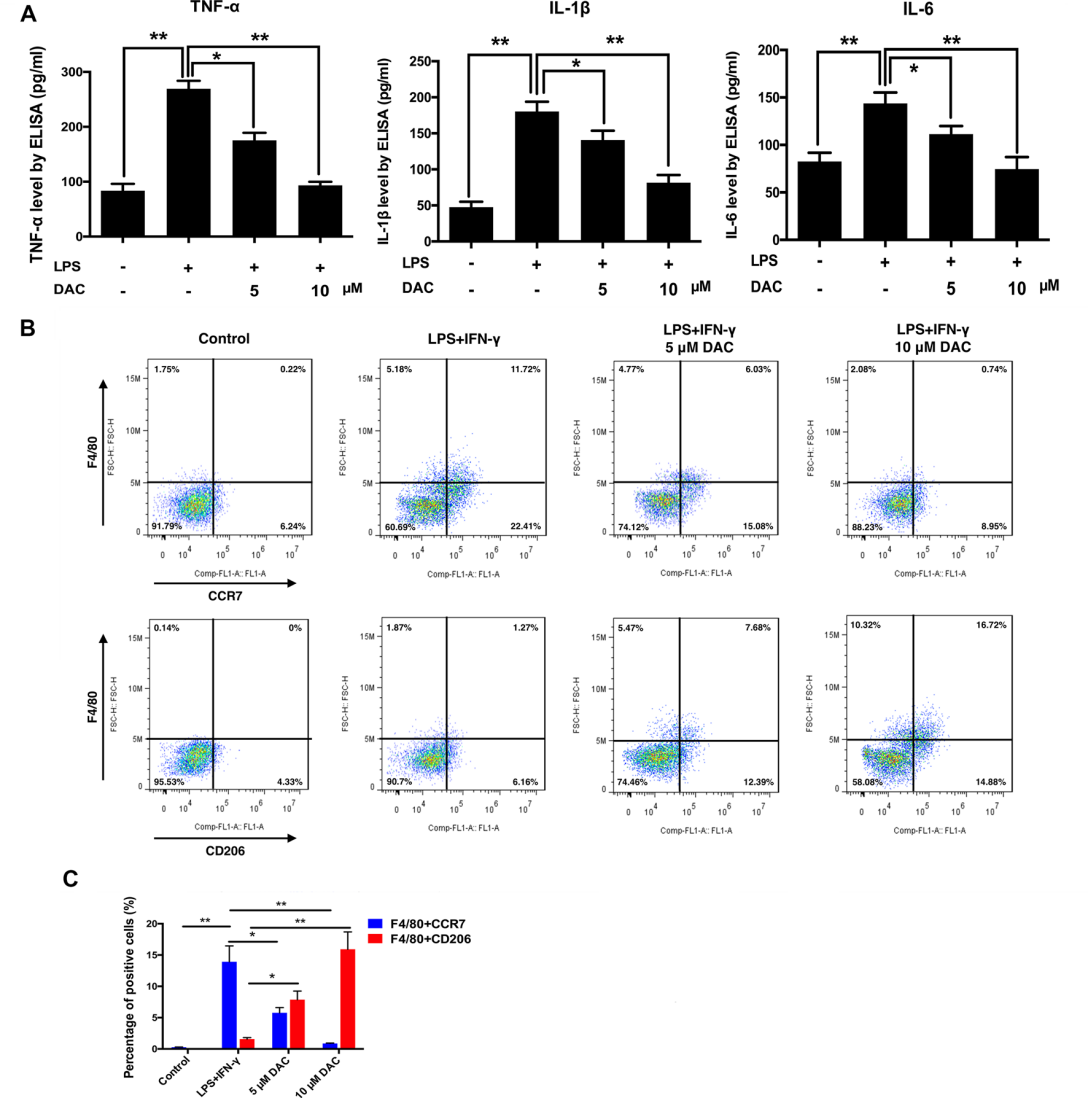
Effects of DAC on polarization of macrophages were detected by flow cytometry. **(A)** The levels of secreted TNF-α, IL-1β, and IL-6 in the medium of cultured calvaria were measured by ELISA. **(B)** Representative histograms of flow cytometry for CCR7 and CD206 as markers of M1 and M2, respectively. **(C)** Percentages of CCR7- and CD206-positive cells were determined by flow cytometry. Results were analyzed using ANOVA followed by Dunnett’s test for *post hoc* analysis. *p < 0.05, **p < 0.01.

A previous study reported that DAC manifests an anti-inflammatory effect (Sosa et al., 2011). Therefore, the secretions of LPS-induced inflammatory cytokines including TNF-α, IL-1β, and IL-6 from BMMs were detected by ELISA *in vitro*. Consequently, the secretions of TNF-α, IL-1β, and IL-6 dramatically increased with the stimulation of LPS compared with control (*p* < 0.01), whereas the treatment of DAC significantly inhibited the secretions of these inflammatory cytokines by a dose-dependent manner (*p* < 0.05) (**Figure 3A**).

### DAC Promoted Macrophage Polarization From M1 to M2

It has been demonstrated that reducing the M1/M2 ratio is a potential therapeutic target in treating postmenopausal osteoporosis (Dou et al., 2018). Therefore, the effect of DAC on the polarization of macrophages was investigated. Flow cytometry was used to analyze the expression of CCR7 (M1 marker) and CD206 (M2 marker) on the BMMs. As a result, the expression of CCR7 on LPS+IFN-γ-stimulated cells was upregulated compared with the control group. Then, the DAC treatment inhibited the increases of CCR7 by a dose-dependent manner (*p* < 0.05). The expression of CD206 only significantly increased in DAC-treated cells (*p* < 0.05) (**Figure 3B and C**).

**Figure 4:**
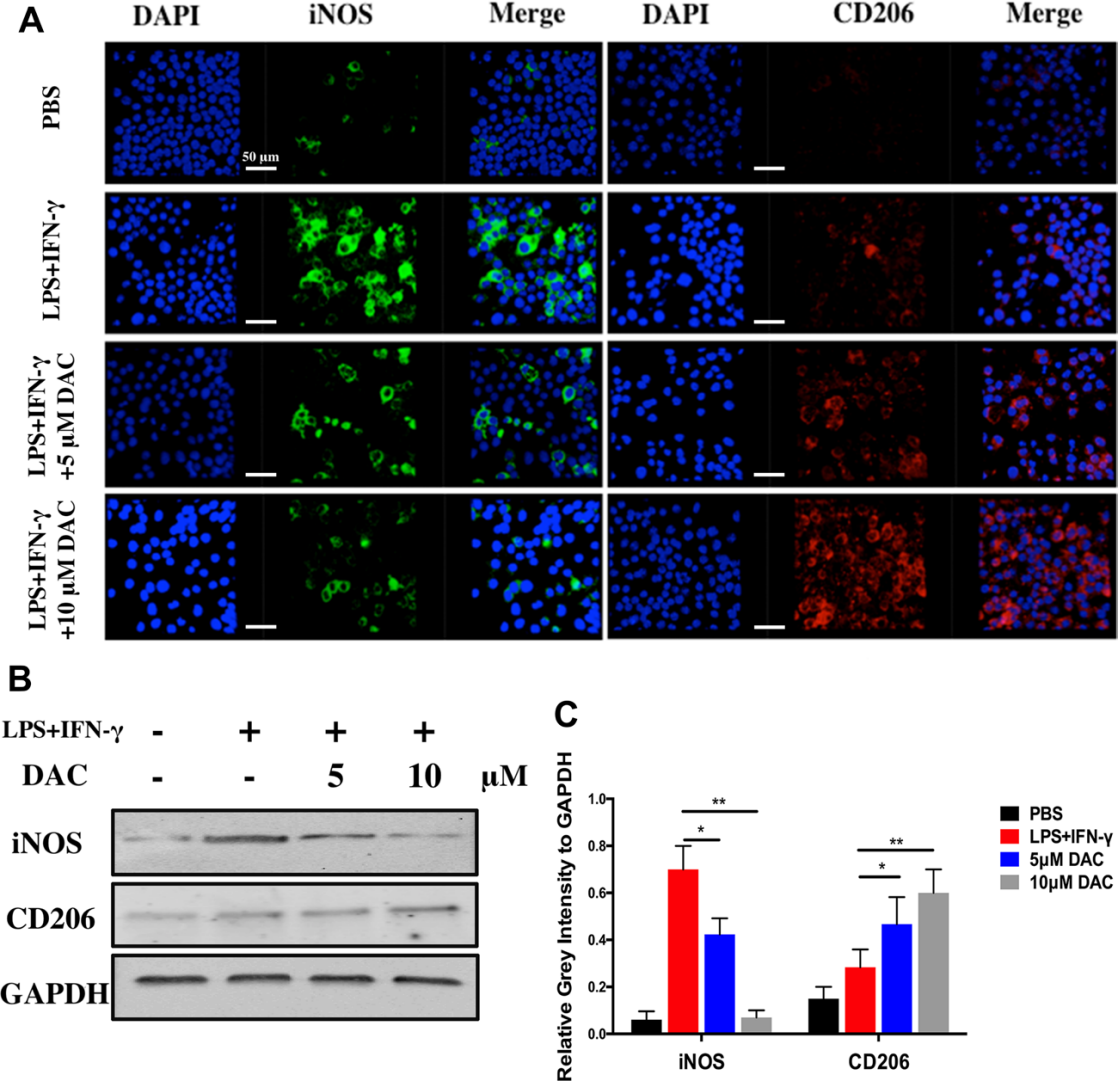
Effects of DAC on polarization of macrophages were detected by immunofluorescence staining. **(A)** iNOS and CD206 expressed in BMMs were detected by immunofluorescence staining. **(B)** The iNOS and CD206 were evaluated by Western blot. **(C)** Relative gray intensity to GAPDH was calculated. Results were analyzed using ANOVA followed by Dunnett’s test for *post hoc* analysis. *p < 0.05, **p < 0.01.

Furthermore, the polarization of macrophages was identified by immunofluorescence staining using anti-iNOS (M1) antibodies, anti-CD206 (M2) antibodies, and DAPI (nuclei) (**Figure 4A**). The expression of iNOS decreased in pre-osteoclasts stimulated with DAC in a concentration-dependent manner (**Figure 4A**, green), whereas CD206 was upregulated in DAC-treated cells compared with control (red). Then, the expressions of iNOS and CD206 in BMMs from different groups were detected by immunoblot. Consistent with the above results, iNOS was upregulated in LPS+IFN-γ-stimulated cells but then inhibited when DAC was added (*p* < 0.05). Accordingly, the expression of M2 polarization marker CD206 only in DAC-treated cells was upregulated compared with control (*p* < 0.05) (**Figure 4B and C**). Collectively, as the concentration of DAC increased, the percentage of M1 macrophages decreased and, in contrast, the ratio of M2 macrophages in the DAC-treated groups was increased, which indicates that DAC reduced the ratio of M1/M2 macrophages as an anti-inflammatory factor.

### The NF-κB and JNK Were Activated During the DAC Inhibiting RANKL-Induced Osteoclastogenesis

**Figure 5:**
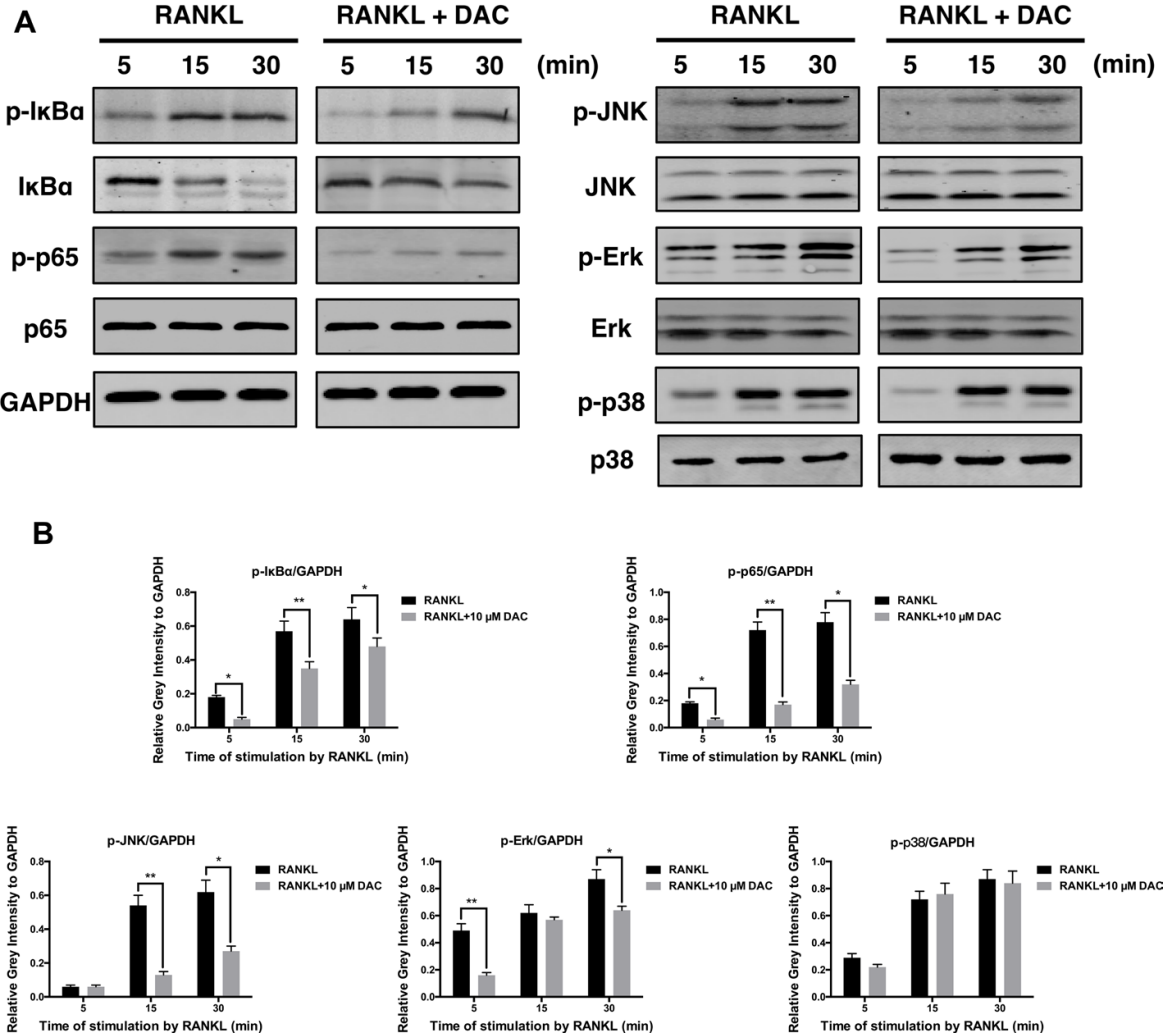
DAC inhibited the activation of NF-κB and MAPK. **(A)** Phosphorylation of IκB, p65, JNK, Erk, and p38, in response to DAC was measured. **(B)** Relative gray intensity to GAPDH was calculated. Results were analyzed using ANOVA followed by Dunnett’s test for *post hoc* analysis. *p < 0.05, **p < 0.01.

For further analyzing the molecular mechanism, the activations of NF-κB and MAPK pathway during the DAC inhibiting RANKL-induced osteoclastogenesis were investigated. As previously reported, the binding of RANKL to its receptor can subsequently activate the downstream kinases including NF-κB and MAPK to upregulate the NFATc1 and c-fos during RANKL promoting osteoclastogenesis (Liu et al., 2019; Xu et al., 2019). Consequently, it was observed that RANKL indeed promoted the phosphorylation of IκBα, p65, JNK, Erk, and p38 in pre-osteoclasts. Then, the activations of p-IκBα, p-p65, p-JNK, and p-Erk were inhibited by DAC, whereas the phosphorylation of p38 was not dramatically affected (**Figure 5A and B**).

### Therapeutic Effects of DAC on the LPS-Induced Murine Calvarial Inflammatory Osteolysis Model

**Figure 6:**
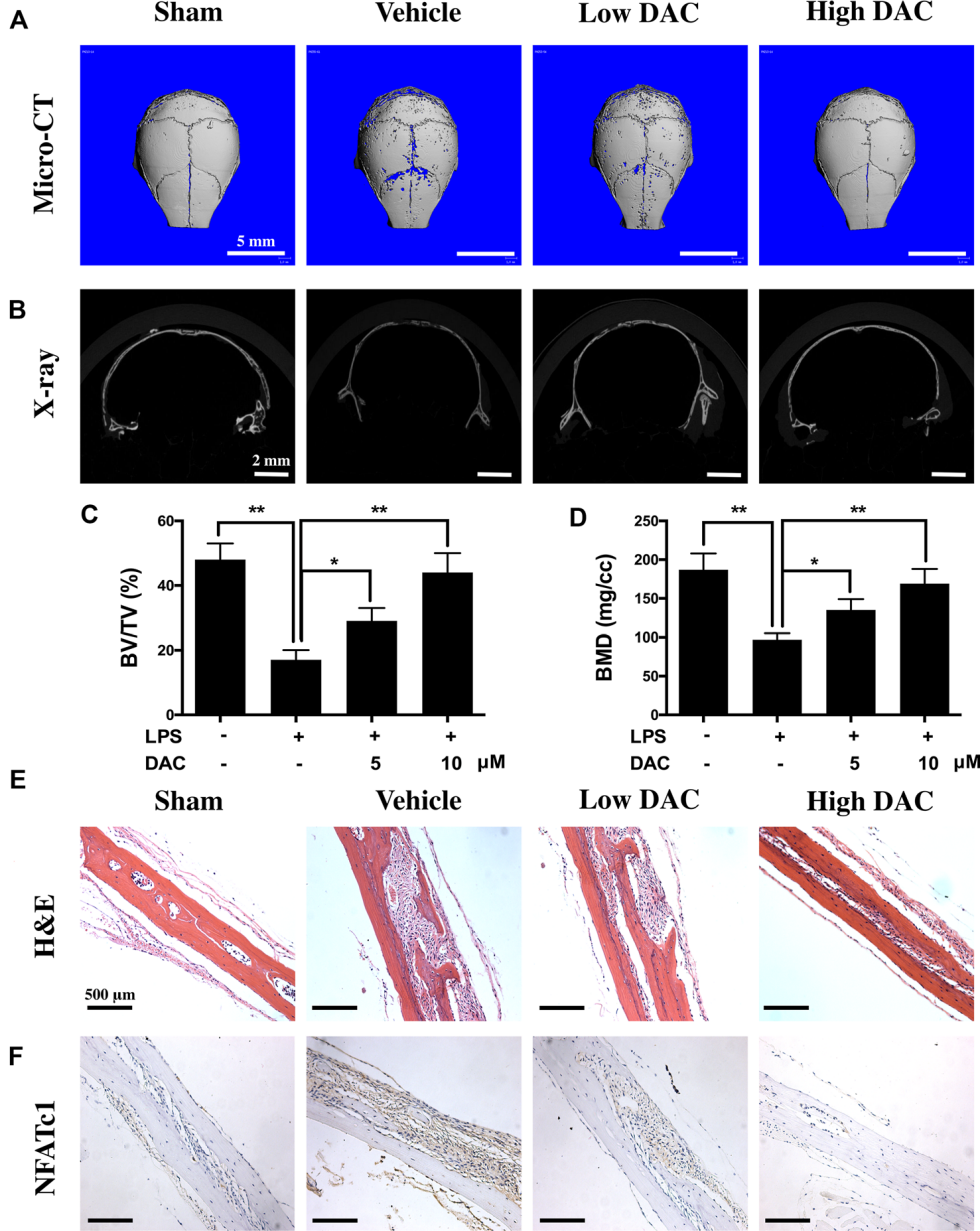
Anti-bone resorption effects of DAC in the LPS-induced mice calvaria osteolysis model. **(A** and **B)** Representative images of mouse calvaria from four groups by micro-CT and X-ray. **(C)** The bone mineral density (BMD) and **(D)** bone volume/tissue volume (BV/TV) were measured. **(E)** H&E staining to histologically evaluate the bone destructions and inflammation. **(F)** NFATc1 was detected by immunohistochemistry. Results were analyzed using ANOVA followed by Dunnett’s test for *post hoc* analysis. *p < 0.05, **p < 0.01.

The effects of DAC on osteoclast-mediated bone resorption were studied using an LPS-induced mouse calvaria osteolysis model. Three-dimensional reconstruction of micro-CT images confirmed that the injection of LPS onto the surface of mouse calvaria effectively induced skull bone inflammatory resorption (**Figure 6A and B**). Further measurements and analyses showed that the BV/TV and BMD of calvaria in the LPS group (i.e., vehicle group) were significantly reduced compared with those in the Sham group (*p* < 0.01, **Figure 6C and D**). In contrast, the treatment of DAC effectively prevented inflammatory osteolysis caused by LPS. Much less resorption pores were observed in DAC-treated groups in comparison to the LPS group. BV/TV and BMD, which were critical indicators of bone mass, also decreased after the administration of DAC in a dose-dependent manner (*p* < 0.05, **Figure 6C and D**).

In addition, H&E staining showed that significant osteolysis appeared and intense inflammatory infiltration of inflammatory cells including lymphocytes and macrophages was present in the mice calvaria of the vehicle group (**Figure 6E**). However, the treatment with DAC effectively prevented bone destructions caused by LPS-induced inflammatory osteolysis. Meanwhile, the expression of NFATc1 in calvaria and surrounding tissues was detected by immunohistochemistry, and it was found that NFATc1 was highly expressed in the vehicle group compared with control. However, consistent with the *in vitro* results, treatment of DAC significantly inhibited the expression of NFATc1 during LPS-induced inflammatory osteolysis (**Figure 6F**). Taken together, the above data confirmed that DAC inhibited the LPS-induced mice calvaria inflammatory bone destructions *in vivo* as well.

## Discussion

In recent years, although much progress has been made in terms of treating osteolysis-related diseases caused by osteoclasts, there are few options for drugs to treat inflammatory bone-loss-related diseases, such as granulosis and rheumatoid arthritis. Therefore, it is necessary to identify better drugs to prevent and suppress inflammation and hyperosteoclastogenesis in such diseases. Importantly, in this study, we found that DAC, as a monomer component of *C. genistoides* or the flowers of *H. lyrata* Bunge with natural biological activity, may prevent and treat Ti particle-induced osteolysis and aseptic loosening of artificial joints. Previous studies have reported that DAC has been used to treat inflammation (Sosa et al., 2011). However, the application of DAC for the prevention and treatment of RANKL-induced osteoclastogenesis and LPS-induced inflammatory osteolysis has not been reported.

In this study, it was observed that DAC significantly suppressed the formation of MNCs from BMMs, which was regarded as mature osteoclasts by a dose-dependent manner (**Figure 1**). Moreover, DAC not only decreased the number of F-actin rings formed in osteoclasts but also inhibited hydroxylapatite resorption *in vitro* by osteoclasts. NFATc1 and c-fos, the critical regulatory factor during differentiation of osteoclasts, were accordingly downregulated by DAC during its inhibiting RANKL-induced osteoclastogenesis (**Figure 2**). Taken together, DAC dramatically inhibited the differentiation as well as the function of osteoclasts *via* decreasing the formation of F-actin rings and hydroxylapatite resorptions in a concentration-dependent manner *in vitro*.

To further investigate the anti-inflammatory property of DAC, the secretions of inflammatory cytokines including TNF-α, IL-1β, and IL-6 from LPS-stimulated BMMs with or without DAC were detected by ELISA. As expected, DAC remarkably decreased the productions of TNF-α, IL-1β, and IL-6 (**Figure 3A**). Additionally, a previous study demonstrated that reducing M1 polarization of macrophage and promoting M2 polarization can inhibit osteoclastogenesis and treat osteoporosis (Dou et al., 2018). In this research, DAC promoted the polarization of BMM cells into M2-type macrophages from M1 in a dose-dependent manner. Our results showed that DAC promoted macrophage polarization from M1 to M2 by increasing the expression of CD206 and inhibiting that of iNOS. Because the morphological and immunostaining results were qualitative, we then quantitatively assessed the percentage of macrophage phenotypes with or without the treatment of DAC using flow cytometry. The LPS and IFN-γ induced the macrophage to M1 polarization with high expression of CCR7, whereas DAC increased the expression of CD206 to transfer the polarization of macrophages to M2 phenotype (**Figure 3**). Meanwhile, the macrophage-specific markers iNOS (M1) and CD206 (M2) were detected to identify phenotypes of macrophages by immunofluorescence analysis (Zhang et al., 2016). Consistent with previous observations, DAC induced a higher percentage of M2 macrophages and reduced the secretion of pro-inflammatory cytokines (TNF-α, IL1-β, and IL-6), characterized by downregulation of inflammation and activation of M2 macrophages (Martinez et al., 2009). Moreover, the iNOS was downregulated and CD206 was upregulated by DAC at the same time, as demonstrated by immunoblotting. In general, *in vitro* results suggested that DAC promoted macrophage differentiation into the M2 phenotype in a concentration-dependent manner to inhibit inflammation.

Further analysis revealed that the inhibitory effects of DAC were related to the NF-κB and MAPK pathways. Specifically, DAC inhibited phosphorylation of JNK, ERK, and p65, resulting in reduction of osteoclastogenesis (**Figure 5**).

As expected, DAC also attenuated LPS-induced osteolysis *in vivo*. The LPS-induced inflammation led to an obvious osteolytic reaction in calvarial bone, as illustrated by extensive bone resorption observed using micro-CT and bone histomorphological analysis. The LPS-induced loss of BMD and BV/TV were significantly rescued in DAC-treated groups. H&E staining illustrated that the destructions of bone were dramatically prevented and inflammatory cells were reduced in DAC-treated groups. The NFATc1 in LPS groups was highly expressed in mouse calvaria and surrounding tissues and inhibited in DAC-treated groups, meaning that osteoclastogenesis was also suppressed *in vivo* (**Figure 6**).

In summary, we found that DAC could effectively protect bones by inhibiting osteolysis. Moreover, it was demonstrated that DAC suppressed RANKL-induced osteoclastogenesis *via* suppressing the phosphorylation of NF-yl and MAPK pathways to inhibit the expression of downstream genes, including c-Fos and NFATc1. Furthermore, DAC modulated the polarization of macrophages from M1 to M2 phenotype, with a shift toward macrophage-specific marker expression and cytokine secretion profiles, which may contribute to its anti-inflammatory properties. These findings provide a basis for further studies of the clinical applications of DAC in the treatment of bone loss-related diseases, such as osteoporosis, osteoarthritis, aseptic loosening after total joint arthroplasty, and osteolytic osteosarcoma.

## Data Availability Statement

All datasets generated for this study are included in the manuscript and/or the supplementary files.

## Ethics Statement

All experiments were approved by the Ethics Committee of Shanghai Tongren Hospital affiliated with Shanghai Jiao Tong University School of Medicine. Animal care and experiments were conducted in accordance with guidelines and procedures authorized by the Animal Care and Use Committee of Shanghai Jiao Tong University School of Medicine.

## Author Contributions

ZL and WX designed this research project. RX, ZL, and XZ performed the cellular and biological molecular experiments. Meanwhile, YW, ZL, and RH performed animal experiments. RX and WX collected and analyzed all data. Eventually, WX wrote the manuscript.

## Funding

The study was supported by the Excellent young medical talents training plan of the Shanghai Health Planning Commission (2018YQ46) and K.C. Wong Education Foundation, Hong Kong.

## Conflict of Interest Satement

The authors declare that there is no conflict of interest in this study.

## Abbreviations

iNOS, inducible nitric oxide synthase; NFATc1, nuclear factor of activated T-cells 1; JNK, c-Jun N-terminal kinase; TJA, total joint arthroplasty; PMMA, polymethyl methacrylate; UHMWPE, ultra-high-molecular-weight polyethylene; NF-κB, nuclear factor kappa B; TNF-α, necrosis factor-α; RANKL, receptor activator of nuclear factor kappa-B (NF-κB) ligand; MAPK, mitogen-activated protein kinase; M-CFS, macrophage-colony-stimulating factor; TRAP, tartrate-resistant acid phosphatase; BMM, bone marrow macrophages; GAPDH, glyceraldehyde 3-phosphate dehydrogenase; CTK, cathepsin K; CTR, calcitonin receptor; DC-STAMP, dendrocyte-expressed seven-transmembrane protein; OSCAR, osteoclast-associated, immunoglobulin-like receptor.
